# Mitochondrial Function and Dysfunction in Dilated Cardiomyopathy

**DOI:** 10.3389/fcell.2020.624216

**Published:** 2021-01-12

**Authors:** Daniela Ramaccini, Vanessa Montoya-Uribe, Femke J. Aan, Lorenzo Modesti, Yaiza Potes, Mariusz R. Wieckowski, Irena Krga, Marija Glibetić, Paolo Pinton, Carlotta Giorgi, Michelle L. Matter

**Affiliations:** ^1^University of Hawaii Cancer Center, Honolulu, HI, United States; ^2^Department of Medical Sciences, University of Ferrara, Ferrara, Italy; ^3^Laboratory of Technologies for Advanced Therapy (LTTA), Technopole of Ferrara, Ferrara, Italy; ^4^Laboratory of Mitochondrial Biology and Metabolism, Nencki Institute of Experimental Biology of Polish Academy of Sciences, Warsaw, Poland; ^5^Center of Research Excellence in Nutrition and Metabolism, Institute for Medical Research, University of Belgrade, Belgrade, Serbia; ^6^Maria Cecilia Hospital, GVM Care & Research, Cotignola, Italy

**Keywords:** mitochondria, cardiomyocytes, cardiomyopathies, organoids model, sarcoplasmic reticulum, Ca ATPase (SERCA) 2+, calcium, heart function

## Abstract

Cardiac tissue requires a persistent production of energy in order to exert its pumping function. Therefore, the maintenance of this function relies on mitochondria that represent the “powerhouse” of all cardiac activities. Mitochondria being one of the key players for the proper functioning of the mammalian heart suggests continual regulation and organization. Mitochondria adapt to cellular energy demands via fusion-fission events and, as a proof-reading ability, undergo mitophagy in cases of abnormalities. Ca^2+^ fluxes play a pivotal role in regulating all mitochondrial functions, including ATP production, metabolism, oxidative stress balance and apoptosis. Communication between mitochondria and others organelles, especially the sarcoplasmic reticulum is required for optimal function. Consequently, abnormal mitochondrial activity results in decreased energy production leading to pathological conditions. In this review, we will describe how mitochondrial function or dysfunction impacts cardiac activities and the development of dilated cardiomyopathy.

## Introduction

Mitochondria are highly dynamic organelles, universally recognized as the “powerhouse” of eukaryotic cells, especially in those that require high-energy demand such as cardiomyocytes (Nan et al., 2017). In these cells mitochondria occupy 30% of the total volume of the cell and supply, through oxidative phosphorylation (OXPHOS), approximately 6 kg of adenosine triphosphate (ATP) per day that is required to sustain cardiac function (Cao and Zheng, 2019). In addition to their pivotal role in energy production, mitochondria are the central hub of cellular metabolism providing metabolites for biosynthesis and also producing reactive oxygen species (ROS). Under physiological conditions ROS act as second messengers that are maintained at low concentrations by the scavenging system present in the cell. However, ROS are hyper-produced in many cardiovascular diseases (CVDs), which impairs heart function (Murphy et al., 2016).

It is well established that mitochondrial calcium (Ca^2+^) fluxes are a key regulator of cardiac function, controlling not only ATP production and mitochondrial metabolism, but also playing a pivotal role in the modulation of muscle contraction (Walsh et al., 2009). In cardiomyocytes mitochondria are well organized and in close proximity to the sarcoplasmatic reticulum (SR), where most cellular Ca^2+^ is stored (Frederick and Shaw, 2007). Therefore, mitochondria are highly sensitive to Ca^2+^ oscillations. The release of Ca^2+^ from SR to mitochondria ensures a balanced activation of SR ATPase and mitochondrial ATP synthesis; all of which contribute to controlling the energy metabolism within a cell (Balaban et al., 2003). Hence, the maintenance of Ca^2+^ homeostasis is a fundamental requirement for optimal mitochondrial function as mitochondria are a key checkpoint regulating cell survival and cell death.

It is thus not surprising that the maintenance of efficient inter-organelle-communication as well as a conserved “mitochondrial quality control” system (MQC), are fundamental for sustaining mitochondrial bioenergetics demand and metabolic functions (Campos et al., 2016). The term MQC refers to mitochondrial fusion and fission machinery (also called mitochondrial dynamics) and autophagy (called mitophagy when pertaining to mitochondria) (Fan et al., 2020). As we will explain in detail in this review, mitochondrial fusion has the ability to respond to high-energy demand conditions by recovering mitochondria that have been damaged and creating elongated interconnected mitochondrial networks. Fission, however, is the process by which dysfunctional mitochondria are separated and segregated away from healthy ones. These dysfunctional mitochondria may be subsequently either recovered or eliminated through mitophagy (Murphy et al., 2016; Fan et al., 2020; Forte et al., 2020; Oh et al., 2020). These complex processes provide the balance for maintaining proper mitochondrial dynamics through regulation of mitochondrial size, shape and number (Youle and Karbowski, 2005; Piquereau et al., 2013).

An increasing number of studies on cardiac mitochondria have determined that dysfunction in their structure and function contributes to the pathogenesis of CVD including dysrhythmias, ischemia-reperfusion injury (IRI) and cardiomyopathies (CMPs); all of which culminate in end-stage heart failure (HF) (Brown and O’Rourke, 2010; Cadenas et al., 2010; Rosca and Hoppel, 2010; Verdejo et al., 2012).

In this review, we will provide an overview of the main functions of mitochondria within cardiac tissue. Furthermore, we will discuss the involvement of mitochondrial impairment in CVD, focusing our attention on dilated cardiomyopathy (DCM) leading to heart failure. Dilated cardiomyopathy is associated with decreased mitochondrial biogenesis and we will examine DCM subtypes and how mitochondria are dysregulated in these conditions. We will highlight the paucity of targeted treatments for DCM and the necessity for understanding the molecular mechanisms involved in DCM onset and progression. Finally, we will the need for new methods to tease out the complexities of dilated cardiomyopathy, such as the potential use of cardiac organoids to investigate the underlying molecular mechanisms of cardiac function and to develop new targeted therapies for dilated cardiomyopathy.

## Mitochondrial Functions in the Heart

### Bioenergetics, Ca^2+^ Homeostasis, Cell Death

**FIGURE 1:**
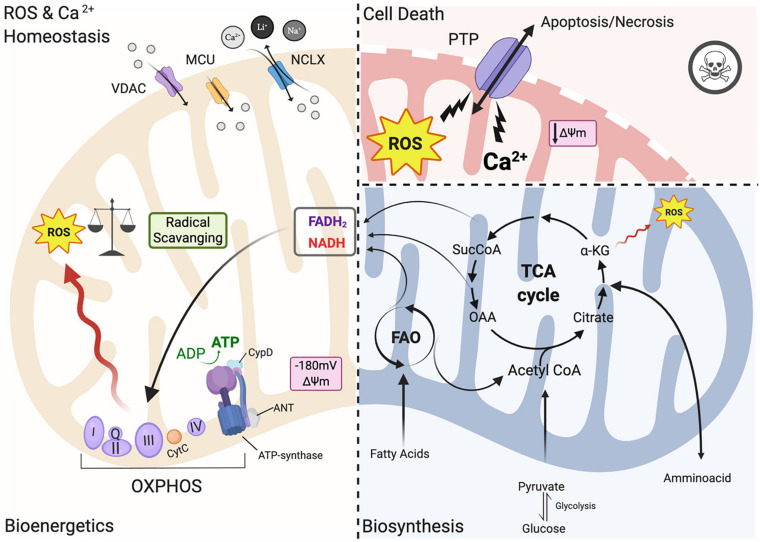
Mitochondrial functions. ***Left panel:*** under physiological conditions, mitochondria functions are the core of bioenergetics activities, providing ATP throughout the OXPHOS, which is also an important source of ROS. Basal ROS levels are maintained by the radical scavenging network. Additionally, mitochondria are calcium-buffering organelles. Ca^2+^ homeostasis is finely controlled by its uptake through voltage-dependent anion-selective channel proteins (VDACs) and the mitochondrial Ca^2+^ uniporter (MCU) complex, Ca^2+^ efflux is controlled by NCLX. ***Right top panel*:** pathological conditions, ROS burst and mitochondrial Ca^2+^ overload activate regulated cell death (RCD) inducing either apoptosis or necrosis pathway through the PTPC opening. ***Right-bottom panel*:** Ca^2+^ uptake activates mitochondrial metabolism. Fatty acids are metabolized via FAO toward the TCA cycle providing energy as FADH_2_ and NADPH are building blocks for biosynthesis. Voltage-dependent anion-selective channel proteins (VDAC), Mitochondrial Calcium Uniporter Complex (MCUC), Mitochondrial Na^+^/Ca^2+^ exchanger (NCLX), oxidative phosphorylation (OXPHOS), Permeability transition pore complex (PTPC), ADP/ATP translocase (ANT) and peptidyl-prolyl *cis-trans* isomerase Cyclophilin D (CypD), cytochrome C (cyt C), adenosine triphosphate (ATP), reactive oxygen species (ROS), tricarboxylic acid cycle (TCA), fatty acid oxidation (FAO), α -ketoglutarate dehydrogenase (α-KG), oxaloacetate (OAA), Acetyl coenzyme A (Acetyl CoA) mitochondrial membrane potential (Δψ_*m*_) (Created with BioRender.com).

In heart mitochondria, the primary source of carbons for ATP production relies on fatty acid oxidation (FAO) (Figure 1). Products of beta-oxidation are directed into the tricarboxylic acid cycle (TCA): the starting compound acetyl-CoA enters the cycle and undergoes a series of reactions where electrons are extracted from TCA intermediates in the form of the reducing equivalents NADH and FADH_2_ and in turn fueling the electron transport chain (ETC) for ATP synthesis (Murphy et al., 2016; Martínez-Reyes and Chandel, 2020). The ETC creates an electrochemical gradient (ΔΨm is −180 mV) along the intermitochondrial membrane (IMM) interface which acts as a driving force for mitochondrial Ca^2+^ uptake (Giorgi et al., 2012, 2018b; Figure 1). Mitochondria are calcium-buffering organelles in which under resting conditions mitochondrial Ca^2+^ concentrations are kept low, but after a stimulus Ca^2+^ is transferred from the SR into the mitochondria that transiently and rapidly takes up large quantities of Ca^2+^ (Giorgi et al., 2018a,b). Lastly, Ca^2+^ is extruded from mitochondria by the Na^+^/ Ca^2+^ antiporter (NCLX) (Giorgi et al., 2018a; Figure 1).

However, under pathological conditions, a cytosolic Ca^2+^ overload initiates a large and persistent Ca^2+^ uptake by mitochondria, which triggers the opening of the mitochondrial permeability transition pore (mPTP; a nonspecific pore) (Figure 1; Giorgi et al., 2012; Morganti et al., 2018). mPTP allows for free passage of small molecules and ions (<1.5 kDa) across the IMM, leading to membrane potential dissipation, and consequent imbalance in ATP production, mitochondrial swelling and mitochondrial outer membrane (OMM) rupture all of which cause regulated cell death (RCD) through either apoptosis or necrosis (Figure 1; Bonora et al., 2015). The activation of one of these cell death pathways depends upon the severity of the damage and the kinetics of the pore opening (Javadov and Karmazyn, 2007). mPTP is also involved in the pathogenesis of Ischemia/reperfusion injury (IRI) (Morciano et al., 2015). For example, a moderate injury, which occurs in the case of a short ischemic period, may lead to a short pore opening time thereby triggering apoptosis. On the other hand, a more severe and persistent insult such as a longer hypoxic event may lead to persistent pore opening inducing cell death through necrosis. Myocardial infarction exhibits a necrotic area at the core of the ischemic zone that is surrounded by apoptotic markers, indicating decreased cardiomyocyte survival upon an ischemic event (Javadov and Karmazyn, 2007; Morciano et al., 2015). The mPTP structure remains an area of intense study; the latest findings have been reviewed recently by Bonora et al. (2020).

In the past few years, the mitochondrial F1/F0 ATP Synthase (ATP synthase) has been recognized as a key component of pore formation along with ADP/ATP translocase (ANT) and peptidyl-prolyl *cis-trans* isomerase Cyclophilin D (CypD) (Figure 1; Bonora et al., 2017; Morciano et al., 2017; Bonora and Pinton, 2019) that together regulate the opening of the permeability transition pore complex (PTPC) (Bonora et al., 2020). It has been demonstrated that dissociation of ATP synthase dimers upon mitochondrial permeability transition (MPT) induction, in particular the C subunit of the F0 part (in its c-ring form), is a key component of the pore (Bonora et al., 2013, 2015, 2017). Mitochondria isolated from *Ppif*-null mice strongly validates the role of CypD as a pore regulator (Basso et al., 2005) because these mitochondria are unresponsive to mitochondrial Ca^2+^ overload. Moreover, upon knocking out all three ANT isoforms simultaneously MPT is inhibited (Karch et al., 2019). It remains controversial whether ANT represents a key pore regulator or is a part of the pore, and futher studies are needed to understand its role in PTPC opening (Bonora and Pinton, 2019).

Thus, in order to avoid mitochondrial Ca^2+^ overload and consequent activation of regulated cell death (RCD), Ca^2+^ uptake has to be finely controlled. Ca^2+^ released by the ER rapidly enters the mitochondrial intermembrane space (IMS) by Voltage-dependent anion channels (VDACs), which are localized at the OMM (Figure 1; Giorgi et al., 2018b). The channel exists in three isoforms (VDAC1, VDAC2, VDAC3) expressed almost ubiquitously among tissues with different sub-mitochondrial ratios (Messina et al., 2012). It exhibits two conformations: the open pore conformation with a low transmembrane potential, showing high-conductance and weak anionselective; whereas increasing potential leads to a closed state conformation characterized by cation selectivity and impermeable to nucleotide passage (Giorgi et al., 2018b). In recent years, the mitochondrial Ca^2+^ uniporter (MCU) was identified as a major player in the regulation of mitochondrial Ca^2+^ homeostasis (Figure 1; Kirichok et al., 2004). MCU is a multiprotein complex (MCUC) situated at the IMM, which has a low affinity for Ca^2+^ ions but is highly selective and regulated by auxiliar proteins that make up part of the MCUC (Marchi and Pinton, 2014). MCUC involvement in cardioprotection has been widely studied in recent years: several mouse models with MCU deletions have been developed including a cardiac-specific dominant-negative MCU mouse that is expressed in neonates (Rasmussen et al., 2015), a cardiac conditional MCU-KO mouse (Luongo et al., 2015), and a tamoxifen-inducible cardiac-specific loss of MCU in adult mice (Kwong et al., 2015). Mitochondria isolated from the hearts of these mice are characterized by reduced mitochondrial Ca^2+^ influx with subsequent reduced susceptibility to mPTP opening and loss of mPTP-related cardioprotection (Kwong et al., 2015; Luongo et al., 2015). On the contrary, deletion of NCLX, a key component of Ca^2+^ release, is lethal to cells because it induces mitochondrial Ca^2+^ overload and consequent PTP opening (Luongo et al., 2015).

### Mitochondria and Biosynthesis

Mitochondria contribute to cell metabolism by providing building blocks for the synthesis of macromolecules necessary for the maintenance of cellular homeostasis and cell growth. As mentioned above, the TCA cycle represents a metabolic engine in mitochondria where these catabolic and anabolic reactions intersect (Martínez-Reyes and Chandel, 2020). As the cycle runs, metabolic intermediates may be utilized for different biosynthetic reactions (Figure 1; Spinelli and Haigis, 2018). These biosynthetic reactions not only consume the TCA cycle intermediates and direct them away from ATP production but also require substantial energy input (Ritterhoff et al., 2020). Thus, whether the intermediates will be used for synthetic purposes is dependent on the energy state of the cell. Energy requirements for sustained cardiac contractile function are high and most of the cardiac metabolism is directed toward the production of ATP (Doenst et al., 2013; Ritterhoff and Tian, 2017; Figure 1). Conversely, biosynthetic demands in non-proliferative cardiomyocytes of the adult heart are rather low, especially compared to highly proliferative cells such as cancer cells (Karlstaedt et al., 2018). However, biosynthesis increases considerably during cardiac hypertrophy (Karlstaedt et al., 2018; Ritterhoff et al., 2020).

Whenever metabolic intermediates are removed from the TCA cycle for biosynthetic reactions, they need to be restored to ensure the cycle’s continued running (Martínez-Reyes and Chandel, 2020). This replenishment of the intermediate pool is named anaplerosis. Increased anaplerotic flux through carboxylation of glycolysis-derived pyruvate to malate was previously reported in hypertrophied rat hearts and paralleled by elevated expression of malic enzyme, which catalyzes this reaction (Sorokina et al., 2007; Pound et al., 2009). During cardiac hypertrophy, there is an energy source switch from FAO to increased glucose utilization, with a general reduction in oxidative metabolism (Doenst et al., 2013; Ritterhoff and Tian, 2017; Ritterhoff et al., 2020). Taken together, increasing the use of pyruvate for anaplerosis reduces its accessibility for oxidation and may lead to energy inefficiency of the TCA cycle (Sorokina et al., 2007; Pound et al., 2009), which contributes to contractile dysfunction and subsequent heart failure. A better understanding of how these mechanisms are regulated is needed for potential targeted treatments of cardiac dysfunction.

### ROS Generation and Regulation

Mitochondria are one of the important sources of ROS production within most mammalian cells, including cardiomyocytes (Figure 1; Chen and Zweier, 2014). Moreover, interspecies comparisons performed in recent years show that ROS regulatory systems are dependent on organism, type of tissue, physiological state, age and pathological conditions to finely tune the underlying responses (Barja, 1999). The primary ROS generated in cardiac mitochondria is superoxide radical anion (O_2_^⋅⁣–^), which can be reduced through dismutation to hydrogen peroxide (H_2_O_2_). Hydroxyl radicals (OH^⋅^) are also generated from the decomposition of hydroperoxides, or by the reaction of excited atomic oxygen with water. The mitochondrial respiratory chain is a powerful endogenous source of O_2_^⋅⁣–^, which is a toxic by-product of oxidative phosphorylation (Figure 1). Electrons from NADH and FADH_2_ flow through the electron chain to reduce oxygen to form H_2_O (Figure 1). Large amounts of O_2_^⋅⁣–^ are generated when oxygen is incompletely reduced due to electron leaking at complexes I and III (Kussmaul and Hirst, 2006; Bleier and Dröse, 2013; Vinogradov and Grivennikova, 2016). Apart from the sites of ROS production within the mitochondrial respiratory chain there are other mitochondrial enzymes that generate either O_2_^⋅⁣–^ or H_2_O_2_. For example, NADPH oxidase 4 (Nox4) is an important source of ROS in heart mitochondria. Nox4 expression is upregulated in failing cardiomyocytes and contributes to the increase of mitochondrial O_2_^⋅⁣–^ levels that drives oxidative stress (Kuroda et al., 2010). α-ketoglutarate dehydrogenase (α-KGDH) is one of the TCA enzymes that is the most vulnerable to environmental changes (Figure 1; Tretter, 2004). α-KGDH generates O_2_^⋅⁣–^ during its catalytic function upon excessive NADH levels (Tretter and Adam-Vizi, 2005), making α-KGDH an important mitochondrial site for ROS production.

The ROS scavenging network coordinately works to maintain proper basal ROS levels and redox signaling in cells to control mitochondrial oxidative stress (Figure 1). The mitochondrial antioxidant defense system includes endogenous antioxidant enzymes such as superoxide dismutase, catalase, glutathione peroxidase, glutathione reductase, and the peroxiredoxin/thioredoxin system (discussed in greater detail in Peoples et al., 2019). ROS are not only byproducts of mitochondrial metabolism (Figure 1), but are commonly involved as second messengers in cellular signaling impacting both adaptive and maladaptive cardiomyocytes responses (Cave et al., 2006). The redox-sensitive cellular processes are involved in cardiac development and differentiation, angiogenesis, cardiac regeneration and cardiomyocyte apoptosis (Tretter, 2004). Indeed, basal levels of ROS are required for human embryonic stem cells (ESCs) to differentiate into cardiomyocytes (Ji et al., 2010). In particular, accumulating evidence points to mitochondrial-mediated ROS generation as having a key role in cardiomyocyte differentiation. Nox4-dependent mitochondrial oxidative stress is one of the major pathways activated in undifferentiated ESCs, driving their differentiation into cardiomyocytes (Murray et al., 2013). Accordingly, Nox4 depletion in ESCs impairs cardiomyocyte differentiation (Li et al., 2006). Another study demonstrated Nox4 expression was significantly reduced in differentiated cardiomyocytes (Crespo et al., 2010).

Oxidative stress signaling also orchestrates angiogenesis in cardiomyocytes through Nox4 regulation. Nox4 is involved in the stimulation of angiogenesis, protecting against contractile dysfunction and hypertrophy under situations of chronic load-induced stress (Zhang et al., 2010). Furthermore, functional alterations of mitochondria and the subsequent increase of ROS production are critical in cardiac repair and regeneration. Postnatal heart maturation is associated with the transition from glycolytic to oxidative metabolism, which drives an increase in ROS production derived from the ETC and reduces cardiomyocyte regeneration capacity (Puente et al., 2014). In general, ROS levels are increased in response to heart damage including ischemic injury (Chouchani et al., 2014; Puente et al., 2014). Blocking ROS production and activation of scavenger systems promotes heart regeneration after cardiac injury (Tao et al., 2016). Conversely, mitochondrial dysfunction and the subsequent increase of mitochondrial ROS cause cardiomyocytes cell cycle arrest and activates apoptotic responses resulting in lethal dilated cardiomyopathy (Yang et al., 2018; Zhang et al., 2018). Overall, further insight into cellular mechanisms by which mitochondrial redox signaling disturbs physiological oxidative stress may uncover novel CVD therapeutic targets.

## Physical and Functional Communication

In adult cardiomyocytes mitochondria mobility is limited with mitochondria moving along microtubule networks (Frederick and Shaw, 2007). In most mammalian cells mitochondria generally cluster around the nucleus (Yoon, 2004), but mitochondria can be at different cytoplasmic locations leading to mitochondrial heterogeneity within different cell types (Kuznetsov et al., 2009; Piquereau et al., 2013). In cardiomyocytes, this heterogenous population can be divided up into three separate populations, characterized by their location within the cardiomyofibers: subsarcolemmal mitochondria (SSM), intermyofibrillar mitochondria (IFM) or perinuclear mitochondria (PNM) (Shimada et al., 1984). Electron microscopy and transmission electron microscopy show these distinct populations of mitochondria as the morphology is unique for both their location and function (Shimada et al., 1984; Manneschi and Federico, 1995; Vendelin et al., 2005; Wikstrom et al., 2009). SSM are located just under the surface sarcolemma and possess closely packed cristae. Holmuhamedov and colleagues characterized and assessed SSM, finding that SSM have a high sensitivity to Ca^2+^ overload-mediated inhibition of ATP synthesis (Holmuhamedov et al., 2012). PNM are clustered at nuclear pores between and around the two nuclei commonly found in cardiomyocytes. Due to their well-developed curved cristae, PNM have little matrix area that allows for higher ATP production (Hackenbrock, 1966). Lu and coworkers provide us with one of the few studies on PNM and found that PNM morphology is more spherical than IFM and SSM, where the lack of myofibrillar constraints allows for the PNM spherical shape and its high mobility. This group also determined that PNM are physically closer to protein synthesis sites for perinuclear mitochondrial biogenesis, indicating that PNM are involved in transcription and translation processes (Piquereau et al., 2013; Lu et al., 2019). Lastly, IFM are very well organized, as they lay closely parallel to contractile myofilaments. This highly organized structure may cause IFMs to be restricted in their position and mobility; however it also provides bioenergetic support needed for contraction and mitochondrial interaction with the cytoskeleton and the SR (Wilding et al., 2006). IFM form an interface with the SR, which allows molecules to be transported between the SR and mitochondria for effective signal transduction (Eisner et al., 2013).

**FIGURE 2:**
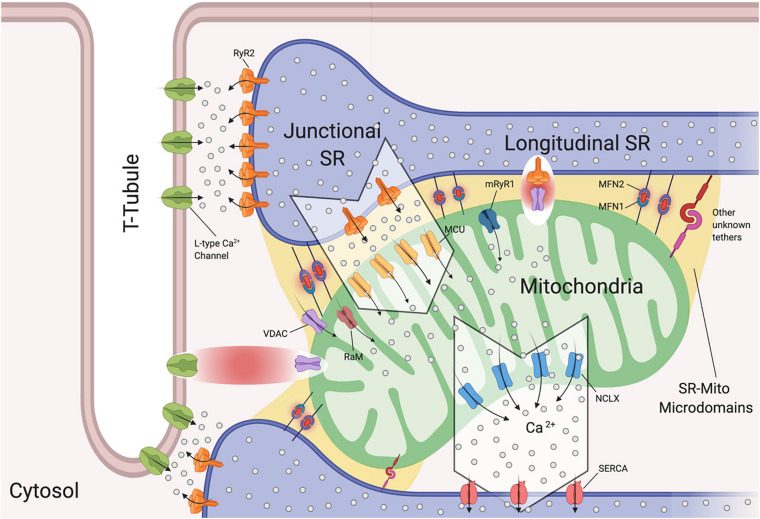
Sarcoplasmic reticulum-mitochondria communications. Sarcoplasmic reticulum (SR) is in close proximity to T-tubules and mitochondria. Depicted are the channels involved in Ca^2+^ flux by which SR regulates excitation-contraction coupling and Ca^2+^ signaling with mitochondria. Microdomains where Ca^2+^ exchanges occur are shown. Ryanodine Receptor 2 (RyR2) and L-Type Ca^2+^ Channels located at the t-tubule-SR interface; voltage-dependent anion-selective channel proteins (VDAC) colocalize with RyR2 and also with L-Type Ca^2+^ channels, Mitochondrial Calcium Uniporter Complex (MCUC) is mainly located at the SR-Mito interface, Rapid modes of Ca^2+^ uptake (RaM), ryanodyne receptor type 1 (mRyR1), Mitochondrial Na^+^/Ca^2+^ exchanger (NCLX) is located at the opposite side of MCUC near the sarcoplasmic/endoplasmic reticulum Ca2+ ATPases (SERCA). Mitofusin1/2 (MFN1/2) function as SR-mitochondrial tethers (Created with BioRender.com).

The SR is of extreme importance in cardiomyocytes, as it critically regulates excitation-contraction coupling by releasing its stored Ca^2+^ via the type 2 ryanodine receptor (RyR2) (Fu et al., 2006; Figure 2). To understand the release of Ca^2+^ by the SR, we will discuss the SR compartments. Structurally, the SR is a diverse organelle, consisting of junctional, corbular, and network SR. These components of the SR form a complex tubular network where the network SR is formed by a series of interconnected tubules that are located in the region between the transverse-tubules (T-tubules) (Figure 2). The junctional SR is the domain where specialized junctions are formed with the sarcolemma T-tubules, allowing the SR to bring its ryanodine sensitive Ca^2+^ channels (RyRs) in close range with the sarcolemma voltage-gated L-type Ca^2+^ channels (Franzini-Armstrong and Protasi, 1997; Franzini-Armstrong et al., 1999; Figure 2). The corbular SR expresses RyRs as well, however it does not form junctions with the sarcolemma (Vega et al., 2011). The membrane depolarization, as a result of excitation-contraction (EC), causes the L-type Ca^2+^ channels to open up close to the junctional SR (jSR). This results in a small amount of Ca^2+^ entering the limited cytosolic space that separates the SR and the T-tubule sarcolemma. This increase in Ca^2+^ concentration exceeds the threshold for the RyR2 to be activated through a mechanism of Ca^2+^-induced Ca^2+^ release (CICR) (Figure 2; Fabiato, 1983). This activation of a small number of clustered RyR2 affects the concentration of intracellular calcium, inducing “calcium sparks” (Cheng et al., 1993). When multiple “sparks” are activated by the EC, a rise of intracellular calcium can be detected in the dyadic cleft (Sharma et al., 2000), thereby initiating myocardial contractions.

Interestingly, it has been reported that L-type Ca^2+^ channels regulate mitochondrial functions through actin filaments (Viola and Hool, 2010). Viola and colleagues demonstrated that Ca^2+^ influx through this channel increases superoxide production, NADH levels, metabolism and also mitochondrial membrane potential in a calcium independent pathway (Viola et al., 2009). The cytoplasmic β-subunit of the L-type Ca^2+^ channel is anchored to the actin cytoskeleton. Disruption of this tether decreases Ca^2+^ flux, leading to poor oxygen consumption and ATP production by the mitochondria (Viola et al., 2009). Moreover, this group found that cardiomyocytes isolated from *mdx* mice (a mouse model of Duschennes muscular dystrophy that in patients causes dilated cardiomyopathy) had impaired communication between L-type Ca^2+^ and mitochondria through an alteration in the cytoskeletal network that led to a decrease in metabolic functions (Viola et al., 2014). They were the first to show a physical and functional association between L-type Ca^2+^ and VDAC through F-actin (Viola et al., 2013, 2014; Figure 2). They further reported that *mdx* cardiomyocytes maintain a higher level of resting calcium and L-type Ca^2+^ channel activation plays a role in the observed mitochondrial calcium changes all of which may promote DCM (Viola et al., 2013).

Interactions between the SR and mitochondria play a key role in cardiomyocyte contraction and multiple studies have provided us with evidence of mitochondrial Ca^2+^ uptake, and thus increased mitochondrial Ca^2+^ levels, in response to SR-mediated Ca^2+^ release (Bassani et al., 1992; Negretti et al., 1993; Szalai et al., 2000). As mentioned previously, cardiac mitochondria uptake cytosolic Ca^2+^ through MCUC. However, this channel requires at least a concentration of 2–5 μM of free Ca^2+^ in the SR’s bulk in order to be activated (Kirichok et al., 2004). This Ca^2+^ concentration is reached only within specific microdomains at the SR-mitochondria interface (Figure 2). Specifically, in such extremely structured cells, MCUC is expressed more in areas in close contact with the jSR, which contain Ca^2+^-releasing RyR2 channels (Figure 2; De La Fuente et al., 2016). Moreover, Ca^2+^ fluxes must be tightly regulated. De la Fuente and colleagues demonstrated that MCUC and NCLX are spatially excluded in cardiac mitochondria (Figure 2) in order to optimize Ca^2+^ signals and sustain mitochondrial metabolism required for cardiomyocyte contraction, while also reducing the energy required for mitochondrial membrane potential depolarization (De La Fuente et al., 2016, 2018). It remains controversial whether mitochondrial Ca^2+^ uptake occurs quickly and synchronously with the cytosolic Ca^2+^ fluctuations in a beat to beat model (García-Pérez et al., 2008) or if it increases slowly (Griffiths et al., 1997). The differences between these two models rely on the experimental approach used in terms of probes, stimulation and species (De la Fuente and Sheu, 2019).

It should be noted that at the microdomain level, the molecular bridge permitting Ca^2+^ exchange between SR-Mitochondria is formed by RyR2 and VDAC2 channels (Figure 2; Min et al., 2012). Moreover, in aged cardiomyoctes the physical interaction between RyR2 and VDAC is significantly reduced, leading to lower mitochondrial Ca^2+^ uptake, thereby promoting oxidative stress and energy impairment (Fernandez-Sanz et al., 2014). However, this event is independent of RyR2 and VDAC expression levels and does not correlate with MFN2 levels (Fernandez-Sanz et al., 2014).

It has become widely accepted that mitochondrial dysfunction is associated with heart disease (Bonora et al., 2019). Pathological SR-dependent Ca^2+^ leak through RyR2 channels is involved in excessive mitochondrial Ca^2+^ uptake (Santulli et al., 2015; Ruiz-Meana et al., 2019). Santulli et al. were the first to show using a murine model that a feedback loop exists between SR and mitochondria where Ca^2+^ leak through RyR2 channels causes mitochondrial Ca^2+^ overload and ROS burst that enhances Ca^2+^ leak and thereby worsening mitochondrial dysfunction (Santulli et al., 2015). Moreover, in human and murine senescent cardiomyocytes, the SR-mitochondria Ca^2+^ exchange is significantly impaired due to RyR2 glycation (Ruiz-Meana et al., 2019). These aged cardiomyocytes display a deficient dicarbonyl detoxification pathway initiating Ca^2+^ leak through RyR2 channels and further increasing mitochondrial Ca^2+^ uptake. Taken together, this mechanism is involved in the transition from a healthy cardiomyocyte to a failing cardiomyocyte, as it may induce bioenergetic deficit through mitochondrial damage leading to mitochondrial dysfunction (Ruiz-Meana et al., 2019).

It is important to mention that MCUC is not the only manner in which mitochondrial Ca^2+^ uptake occurs in cardiomyocytes. Three different approaches of *MCU*-knockout mice (global constitutive, cardiac-specific, dominant negative overexpression) have been developed (Pan et al., 2013; Kwong et al., 2015; Luongo et al., 2015). These models demonstrate that MCUC is dispensable for heart function in basal cardiac activity, while under “fight-or-flight” conditions MCU deletion shows inhibition of acute mitchondrial Ca^2+^ uptake. Moreover, these mice are highly protected from mPTP opening during ischemia-reperfusion injury. Therefore, in basal resting conditions, mitochondrial Ca^2+^ influx for maintaining ATP production and cardiac metabolism occurs through other channels such as rapid modes of Ca^2+^ uptake (RaM) (Buntinas et al., 2001) and ryanodyne receptor type 1 (mRyR1) (Beutner et al., 2001, 2005; Figure 2) both of which are located in the IMM. RaM displays a faster Ca^2+^ uptake compared to MCUC (Buntinas et al., 2001), while mRyR1 opens at lower cytosolic Ca^2+^ concentrations (Beutner et al., 2001, 2005).

As discussed above, in cardiomyocytes SR-mitochondria Ca^2+^ transfer occurs mainly in areas of direct physical contact. However, the proteins involved in the tethering have been poorly investigated in the heart. Currently, mitofusin 2 (MFN2) is the protein that has been suggested to tether this physical interaction (Figure 2; Papanicolaou et al., 2011; Chen et al., 2012). It remains a matter of discussion whether MFN2 acts as a tether or a spacer at the ER/SR-mito interface. Two different *MFN2*-KO mouse models have been generated showing opposite results. When the gene deletion is made after birth the distance of SR-mitochondria increased leading to a rise of Ca^2+^ concentration in the SR (Chen et al., 2012). Moreover, isopronenterol stimulation of cardiomyocytes, display an increase in cytosolic Ca^2+^ concentrations and lower mitochondrial Ca^2^ uptake. Of note, this model does not show mitochondrial bioenergetic impairment (Chen et al., 2012). On the other hand, if the gene is deleted at the embryonic stage, there are no differences in the SR-mitochondria distance and therefore, no alterations in Ca^2+^ fluxes. This result may be due to compensatory remodeling (Papanicolaou et al., 2012). Each of these mouse models demonstrates mitochondrial morphology alterations, contractile depression and cardiac hypertrophy in the adults. However, more studies are needed to investigate the role of MFN2 in tethering SR-mitochondria and also to determine whether other proteins may be involved in this tethering (Figure 2).

## Mitochondria Dynamics

### Fusion and Fission

Mitochondrial health is tightly correlated to the ability of these dynamic organelles to move and change their morphology in response to the surrounding environment (Ong et al., 2017). The term “mitochondrial dynamics” refers to fusion and fission processes of mitochondrial structures within a living cell. Mitochondria constantly shape themselves through fusion and fission in response to changes in energy requirements (Hu et al., 2019). The balance between mitochondrial fusion and fission determines the number (biogenesis), morphology and activity of these multifunctional organelles (Marín-García and Akhmedov, 2016). In the heart, rapid responses to body demands depend on this balance of fusion and fission that modulate multiple mitochondrial functions such as energy production, ROS generation, Ca^2+^ homeostasis and cell death (Marín-García and Akhmedov, 2016).

**FIGURE 3:**
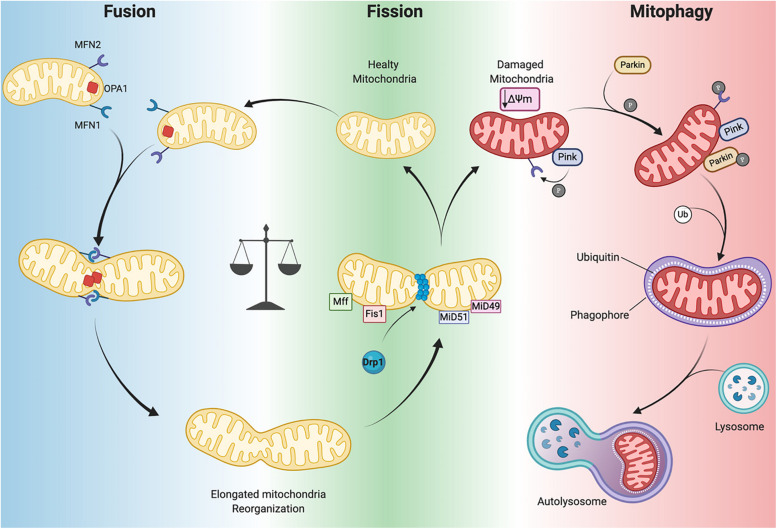
Mitochondrial dynamics. Mitochondrial neworks are mantained by the balance betwen fusion (***left panel***) and fission (***central panel***). Damaged mitochondria are cleared by mitophagy pathway (***right panel***). Dynamin-related protein-1 (Drp1), fission protein-1 (Fis1), mitochondrial fission factor (Mff), mitochondrial dynamics proteins 49 and 51 (MiD49 and MiD51), mitofusin 1/2 (MFN1 and MFN2) optic atrophy 1(Opa1), PTEN-induced kinase 1 (PINK1) ubiquitin ligase Parkin, phosphorylation (P), Ubiquitin (Ub), mitochondrial membrane potential (Δψ_*m*_) (Created with BioRender.com).

Several GTPases are involved in the fission and fusion processes, which utilize GTP energy to guide conformational changes (Urbani and Babu, 2019). Mitochondrial fission is driven by dynamin-related protein-1 (Drp1) that is recruited to mitochondria by human fission protein-1 (hFis1), mitochondrial fission factor (Mff) and mitochondrial dynamics proteins 49 and 51 (MiD49 and 51) (Figure 3; Ong et al., 2017). This process, characterized by the fragmentation of mitochondria into more restricted and rounded organelles (Sharp and Archer, 2015; Sciarretta et al., 2018), is essential for mitosis and is required for the specific clearance of injured mitochondria through mitophagy (Ong et al., 2017). Specifically, in the heart, mitophagy is used to preserve a healthy pool of mitochondria under stress conditions (Nan et al., 2017). Mitochondrial outer membrane fusion is directed by mitofusin 1 (MFN1) and MFN2 and mitochondrial inner membrane fusion is executed by optic atrophy 1(Opa1), leading to the formation of functional elongated organelles (Figure 3; Ong et al., 2017). This process is fundamental for mitochondrial DNA (mtDNA) maintenance and inheritance, membrane potential transmission and Ca^2+^ signaling within the mitochondrial machinery (Westermann, 2010; Archer, 2013; Elgass et al., 2013; Hoppins, 2014). Of note, in stress-free conditions neonatal and adult cardiomyocytes demonstrate differences in the rate of mitochondrial dynamics. Indeed, in neonatal cardiomyocytes fusion and fission processes are more frequent and rapid compared to the levels observed in adult cardiomyocytes (Forte et al., 2020). Mitochondria in neonatal cardiomyocytes are highly mobile and distributed throughout the cytoplasm within a filamentous network, while adult cardiomyocyte mitochondria are more static and spatially arranged into three subpopulations, as described above, which constrains their movements (Vásquez-Trincado et al., 2016). The homeostasis of mitochondrial dynamism is ensured by the interaction and cooperation of the aforementioned proteins. Moreover, alterations in the balance of mitochondrial dynamics are correlated to cardiac disorders (Marín-García and Akhmedov, 2016; Hu et al., 2019) leading to aberrant mitochondrial network morphology.

### Mitophagy

Mitophagy is a specific form of autophagy exploited by the cellular machinery to digest dysfunctional and senescent mitochondria through autophagosomes, under basal and stress conditions (Shires and Gustafsson, 2015). This process is tightly regulated by the mitochondrial PTEN-induced kinase 1 (PINK1) and the cytosolic ubiquitin ligase Parkin. In Parkin-mediated mitophagy, upon loss of ΔΨm, PINK1 accumulates in the OMM where it recruits Parkin (Figure 3). This may occur directly by PINK1-mediated phosphorylation of Parkin (Koyano et al., 2014) or indirectly by phosphorylation of MFN2 (Figure 3; Chen and Dorn, 2013). Indeed, Matsua and colleagues reported that upon a ΔΨm decrease, cytosolic Parkin is recruited to mitochondria by PINK1 through Parkin’s phosphorylation at Ser^65^ within its ubiquitin-like domain. This phosphorylation event is necessary for the efficient translocation of Parkin to mitochondria (an initial step of mitophagy). Upon activation parkin initiates ubiquitination of mitochondrial proteins to promote phagasome recruitment and subsequent degradation of mitochondrial proteins by the lysosome (Figure 3; Kondapalli et al., 2012; Shiba-Fukushima et al., 2012; Koyano et al., 2014).

It has also been observed that the phosphorylation of MFN2 by PINK1 is essential for Parkin recruitment to damaged mitochondria, thus suggesting a connection between mitochondrial dynamics and mitophagy (Figure 3; Chen and Dorn, 2013). A detailed discussion of mitophagy is reviewed elsewhere (Shires and Gustafsson, 2015; Sciarretta et al., 2018). Mitochondrial autophagy plays a critical cardioprotective role; although when impaired it is detrimental to the heart (Saito and Sadoshima, 2015; Morciano et al., 2020). In mouse hearts lacking *Mfn2* expression there is a reduction in Parkin-mediated mitophagy and contractility and increased hypertrophy leading to heart failure by 30 weeks of age (Song et al., 2014). As mentioned previously, during ischemia/reperfusion (I/R) mitophagy appears to protect the heart. Indeed, in a cardiac-specific conditional *Drp1* knockout mouse the inhibition of the mitophagic flux causes accumulation of injured and dysfunctional mitochondria, leading to cardiomyocyte death during reperfusion (Ikeda et al., 2015). In addition, ablation of *Drp1* in adult mouse cardiomyocytes dampens mitochondrial fission and significantly upregulates Parkin, which leads to mitophagy and lethal cardiomyopathy (Song et al., 2015). Notably, loss of Parkin in adult mouse hearts did not affect function. However, in neonates a lethal cardiomyopathy due to defective mitophagy clearance of fetal mitochondria was observed in cardiomyocyte-specific *Parkin* ablation within three weeks after birth (Gong et al., 2015). Moreover, *PINK1* deficiency in mice leads to cardiac mitochondrial dysfunction and excessive oxidative stress (Billia et al., 2011). These findings highlight that PINK1/Parkin and the mitophagy machinery are crucial for cardiac homeostasis. Moreover, mitophagy impairment results in defective cellular homeostasis, leading to cardiomyopathy and ultimately heart failure (Nan et al., 2017; Bonora et al., 2019).

## Dilated Cardiomyopathy and Mitochondrial Dysfunction

Cardiomyopathies are a heterogeneous condition, which effect myocardial structure and function culminating in heart failure. Due to the complexity of this disease its classification continues to evolve. However, the scenario is not simple, since any attempt at classification is limited by the criterion choice of classification itself (phenotype, etiology, clinical, morphological, functional). Additionally, the heterogeneity and all the overlapping forms of cardiomyopathy make this work more complex (Report of the WHO/ISFC task force on the definition and classification of cardiomyopathies; WHO/ISFC, 1980; Maron et al., 2006; Thiene et al., 2007; Arbustini et al., 2014).

Among all cardiomyopathies, dilated cardiomyopathy (DCM) continues to lack proper characterization and understanding (Merlo et al., 2019). It is presented with a mixed etiology and high incidence of genetic mutations. Patients with dilated cardiomyopathy may present with ventricular arrhythmias in the absence of signs of heart failure. An arrhythmogenic indication may be found in arrhythmogenic right ventricular (RV) cardiomyopathy (ARVC) and left-dominant arrhythmogenic cardiomyopathy, hypertrophic cardiomyopathy and LV noncompaction all of which are associated with increased risk of sudden cardiac death. Furthermore, DCM may also occur in patients with mitochondrial cardiomyopathy and metabolic disorders (Thiene et al., 2007; Merlo et al., 2019). Moreover, new genetic mutations continue to be identified for DC, its related peripartum cardiomyopathy and those of the arrhythmogenic cardiomyopathies, including ARVC, left-dominant arrhythmogenic cardiomyopathy, and channelopathies (Sen-Chowdhry et al., 2008, 2010; Spezzacatene et al., 2015).

**FIGURE 4:**
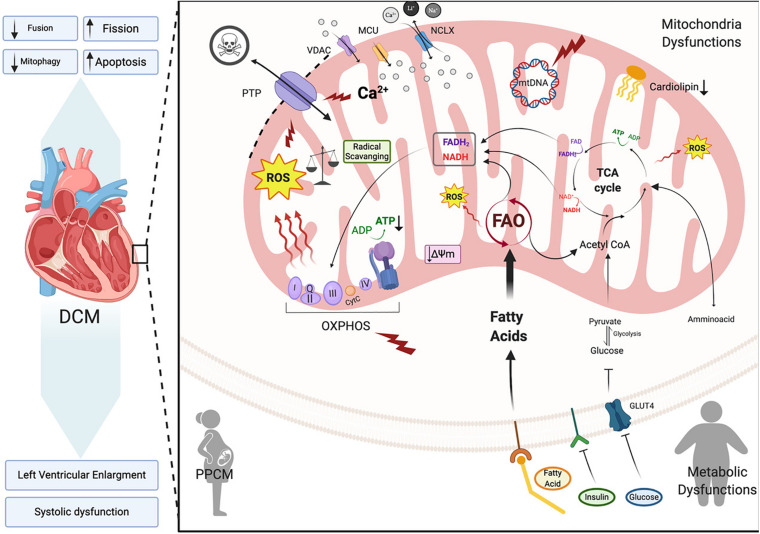
Dilated cardiomyopathy. Schematic representation of the primary pathway of cardiomyocyte mitochondrial dysfunction, which induces dilated cardiomyopathy (DCM). Metabolic imbalance, ROS overproduction and dysregulation of Ca^2+^ homeostasis are key changes inducing dilated cardiomyopathy. These overall changes cause increased mitochondrial fission events and activation of cardiomyocyte apoptosis. Voltage-dependent anion-selective channel proteins (VDAC), Mitochondrial Calcium Uniporter Complex (MCUC), Mitochondrial Na^+^/Ca^2+^ exchanger (NCLX), oxidative phosphorylation (OXPHOS), adenosine triphosphate (ATP), reactive oxigen species (ROS), tricarboxylic acid cycle (TCA), fatty acid oxidation (FAO), Glucose transporter type 4 (GLUT-4), mitochondrial membrane potential (Δψ_*m*_), cytochrome C (cyt C) (Created with BioRender.com).

In this section we will focus on how changes in mitochondrial health are associated with dilated cardiomyopathy onset and progression (Figure 4). Dilated cardiomyopathy characteristics resulting from gene mutations have been identified in patients with peripartum cardiomyopathy, metabolic disorders, mitochondrial dynamics, OXPHOS dysfunction, Fatty acid and cardiolipin metabolism (Barth’s syndrome), all of which we will touch on here (Figure 4). In each of these syndromes echocardiographic data points to dilated cardiomyopathy presenting with left ventricular systolic dysfunction (left ventricular ejection fraction of <45% or fractional shortening of <30%) and cardiac hypertrophy. A number of mitochondrial diseases including OXPHOS disorders (Marin-Garcia et al., 2000; Alston et al., 2012, 2015; Jain-Ghai et al., 2013) and Barth syndrome (Hoch, 1992; Spencer et al., 2006; Gebert et al., 2009; Wang H.-Y.J. et al., 2014) correlate with the incidence of DCM, suggesting mitochondrial function as a key prognostic in the pathogenesis of DCM.

### Dilated Cardiomyopathy Types and Mitochondrial Dynamics

Dilated cardiomyopathy (DCM) is a leading cause of heart failure worldwide with a higher incidence in underdeveloped countries, however its prevalence varies due to geographic and socioeconomic conditions (Bozkurt et al., 2016). It is a form of heart disease that presents with dilation of the left or both ventricles and leads to systolic dysfunction and subsequent heart failure (Figure 4; Dellefave and McNally, 2010; De Paris et al., 2019). DCM is also considered idiopathic if no other vascular conditions such as hypertension are detected (Mestroni et al., 1999). Between 20–35% of idiopathic DCM cases may be linked to a family history with an inherited gene defect (Hershberger et al., 2010). Primarily mutations in genes that are involved in sarcomere structure and contractility, cytoskeletal arrangement, electrolytes balance and mitochondrial function (De Paris et al., 2019) are linked to DCM. In DCM, the cardiac muscle becomes thin and weakened causing the open area of the chamber to become enlarged. As a result, the heart is unable to pump blood efficiently (NIH, 2020). In order to maintain cardiac output, ventricular volumes increase and sarcomere contractility is reduced thereby, producing the thin-walled dilated appearance that characterizes DCM (De Paris et al., 2019). This ventricular remodeling is driven by irregular cardiomyocyte pathophysiology encompassing cardiomyocyte hypertrophy, impaired calcium cycling, apoptosis and fibrosis. A decrease in cardiac efficiency, as measured by myocardial oxygen consumption, leads to a progressive weakening of energy-starved cardiac myocytes pushing the heart toward heart failure (De Paris et al., 2019; Chen, 2020).

Because the heart is an organ requiring a high energy demand, regulation of mitochondrial metabolism plays an important role in the pathogenesis of this and other CVDs (Rosca and Hoppel, 2010). During the first stages of DCM, increased mitochondrial number (Figure 4) acts as a compensating mechanism for maintaining energy supply (Zak et al., 1980). However, the number of mitochondria declines during DCM progression leading to a reduction in ATP, decreased contractility and increased ROS all of which results in diastolic dysfunction and heart failure (Flarsheim et al., 1996; Goffart et al., 2004; Figure 4). Oxidative stress that presents as a consequence of increased ROS production is a key part of the pathogenesis of DCM. *In vitro* studies on isolated rat cardiomyocytes determined that physiologic stretch induces a Ca^2+^ spike through activation of NADPH Oxidase 2 (NOX2) and subsequently the ryanodine receptors in the SR. The authors found that in healthy cardiomyocytes, NOX regulation of ROS production plays a beneficial role through oxidation of the RyR2 channel, which mediates cardiac Ca^2+^-induced Ca^2+^ release (Figure 2). However, in muscular dystrophy *mdx* diseased cardiomyocytes these Ca^2+^ sparks induce arrhythmogenic Ca^2+^ waves (Prosser et al., 2011). Moreover, in an *mdx* mouse model of Duchenne muscular dystrophy Ca^2+^ release led to hyperactive ROS and subsequent cardiomyopathy. Whether this occurs in DCM remains to be determined. However, in patients with DCM, NADPH-upregulation of NOX increases ROS production through elevated rac1-GTPase activity (Maack et al., 2003). Furthermore, isolated ventricular cardiomyocytes from a rabbit heart failure model display decreased Ca^2+^ uptake resulting in reduced mitochondrial NADPH availability (Despa et al., 2002). Decreased NADPH subsequently increases ROS (Kohlhaas et al., 2010; Figure 4). Taken together, these findings point to Ca^2+^ controlled uptake at the mitochondria as a key regulator of ROS and an imbalance in NADPH-ROS causes disturbances in excitation-contraction coupling leading to cardiac dysfunction (Sag et al., 2013) and heart failure. ROS overproduction may also induce myocardial fibrosis, which is a common factor in DCM patients presenting with diastolic and systolic dysfunction (Assomull et al., 2006; Herpel et al., 2006). Cardiac stress, in general, plays a key role in initiating intrinsic apoptotic mechanisms in cardiomyocytes through mitochondrial dysfunction (Green and Reed, 1998). As detailed in previous sections dysfunctional mitochondria are efficiently removed by mitophagy in cardiomyocytes for cell maintenance and survival (Figure 3; Vásquez-Trincado et al., 2016). However, during cardiac stress, autophagy flux is reduced and damaged mitochondria accumulate resulting in enhanced oxidative stress and cardiomyocyte apoptosis (Figure 4; Campos et al., 2016). Uncontrolled autophagy is a component of the pathogenesis of DCM, cardiac hypertrophy and ischemic heart disease (Chistiakov et al., 2018).

### Mitochondrial Fusion and Fission Alterations in Dilated Cardiomyopathy

The role of mitochondrial morphological alterations in the physiopathology of cardiomyopathies have become more apparent in recent years with changes in mitochondrial fission and fusion being at the forefront. Mitochondrial fission and fusion proteins are essential for normal cardiac remodeling and homeostasis (Chen et al., 2011). Impairments in mitochondrial morphology and function due to genetic deletion of fission and fusion proteins and their interacting partners may lead to dilated cardiomyopathy and heart failure (Nan et al., 2017; Ong et al., 2017; Hernandez-Resendiz et al., 2020). Dynamin-related protein 1 (DRP1) is involved in controlling mitochondrial fission. Genetic ablation of the *Drp1* gene is embryonically lethal at day E12.5 (Manczak et al., 2012), however cardiac-specific deletion of *Drp1* in the murine adult heart triggers mitochondrial elongation and mitophagy suppression leading to a higher susceptibility to ischemia/reperfusion and cardiomyopathy (Ikeda et al., 2015). Further studies demonstrated that Python mutant mice with a *Drp1* gene point mutation (C425F) develop mitochondrial defects and DCM, as a result of diminished mitochondrial fission and mitophagy (Cahill et al., 2015). This C452F mutation is within the M domain, which is highly conserved and involved in protein-protein interactions (Cahill et al., 2015). Moreover, mice deficient in the mitochondrial fission regulator *Mff* also develop DCM leading to heart failure and death. Interestingly, the simultaneous deletion of mitochondrial fission and fusion regulator genes *MFF (mitofission)* and *MFN* (mitofusin) rescued *Mff* knockout mice, with improved cardiac function, enhanced mitochondrial oxidative capacity and increased survival (Chen et al., 2015).

In regards to fusion, cardiac-specific ablation of mitofusins *Mfn1* and *Mfn2* in mouse embryos causes death at days E9.5–10.5 (Chen et al., 2011). At late embryonic stage, the genetic inactivation of both these mitofusins promotes mitochondrial dysfunction leading to the development of a lethal cardiomyopathy; possibly due to biogenesis alterations, diminished mtDNA and enhanced mitochondrial fragmentation (Papanicolaou et al., 2012). Furthermore, conditional cardiac *Mfn1/Mfn2* gene deletions in adult mouse hearts present with mitochondrial fragmentation, mitochondrial respiratory chain deterioration and develop a lethal DCM. Interestingly, loss of the *Mfn1* gene alone is well tolerated in mice (Papanicolaou et al., 2011; Chen et al., 2012), whereas *Mfn2*-null mice display mitochondrial enlargement (Chen and Dorn, 2013), increased ROS production (Song et al., 2014) and cardiac hypertrophy (Papanicolaou et al., 2011). Proteolytic processing of fusion protein Opa1 also plays a critical role in the regulation of mitochondrial fusion. Opa1 proteolysis by stress-activated OMA1 peptidase induces mitochondrial fragmentation and DCM onset in a cardiac-specific *Yme1l* peptidase-null mouse (Wai et al., 2015). Taken together, alterations in mitochondrial fusion and fission machinery in the heart promotes mitochondrial metabolic impairments that induce dilated cardiomyopathy.

### Dilated Cardiomyopathy: Subtypes and Syndromes

#### Peripartum Cardiomyopathy

Peripartum cardiomyopathy (PPCM) is a rare form of DCM that develops in the last month of pregnancy or within five months postpartum. It presents with left ventricular systolic dysfunction (left ventricular ejection fraction of < 45% or fractional shortening of <30%) and cardiac hypertrophy (Satpathy et al., 2008). PPCM occurs in the absence of any identifiable cause and is exclusive to patients with no prior history of heart disease (Crane, 1976; Hibbard, 1999). PPCM occurs worldwide with an incidence of 1:1,000 births (Sliwa et al., 2017; Cunningham et al., 2019) with its incidence varying geographically being the highest in the United States, South Africa, Nigeria, and Haiti. In the US, its incidence is 1:2,230 live births (Mielniczuk et al., 2006; Brar et al., 2007; Gunderson et al., 2011; Kolte et al., 2014; Cunningham et al., 2019). Mortality also varies depending location with an estimate of 3–40% of patients diagnosed with PPCM Although it is a condition of unknown etiology, it also occurs in women with a previous history of pre-eclampsia or as a result of multiple pregnancies (Kolte et al., 2014; Arany and Elkayam, 2016).

PPCM shares many similarities with DCM including clinical symptoms such as ventricular dilation and systolic dysfunction (Figure 4; Pearson et al., 2000; Elliott et al., 2007). However, both diseases differ in progression and outcome and there are molecular differences. Similar to DCM, oxidative stress plays a key role in the pathogenesis of PPCM in patients and in mouse models in which ROS levels are highly increased compared to controls. According to Hilfiker-Kleiner and colleagues (Hilfiker-Kleiner et al., 2007), one possible reason for this increase is due to a cardiomyocyte-specific deletion of signal transducer and activator of transcription 3 (STAT3) as studied in a mouse model of PPCM. In these mice downregulation of the antioxidant Manganese superoxide dismutase (MnSOD) increases ROS production within mitochondria, which activates Cathepsin D that cleaves prolactin (PRL, 23 kDa) into a smaller 16 kDa piece. This negatively affects cardiomyocyte microvasculature and metabolism in these mice (Hilfiker-Kleiner et al., 2007, 2012; Hilfiker-Kleiner and Sliwa, 2014). STAT3 levels are downregulated in PPCM patient hearts suggesting that its expression may be cardioprotective during pregnancy (Ricke-Hoch et al., 2013). Importantly, PLR levels increase and remain high toward the end of pregnancy and after delivery which is in accord with the development of PPCM (Grattan et al., 2008). PPCM patients present with changes in PRL as well (Hilfiker-Kleiner et al., 2007; Haghikia et al., 2013) suggesting an oxidative stress plays a key role in PPCM.

A different mouse model of PPCM containing a cardiac specific deletion of PPARγ coactivator-1α (PGC-1α) showed that MnSOD was also reduced in the heart thus increasing ROS production and resulting in disturbed mitochondrial metabolism (Patten et al., 2012). Although research in cardiomyopathy-related genes have begun to elucidate the pathogenesis of PPCM, the molecular mechanisms underlying the development and progression of PPCM and development of targeted therapies have yet to be elucidated.

Early case studies in cardiomyopathy-related genes identified a clinical overlap between PPCM and DCM, however the extent of this interconnection remains unknown (Lee and Judge, 2017). More recent studies have reported that mutations in cardiac sarcomere proteins are a pathogenic cause of PPCM. These mutations include deleterious truncations in the titin encoding *TTN* gene that is a clinical feature shared with idiopathic DCM (van Spaendonck-Zwarts et al., 2014; Ware et al., 2016; Ballard, 2019). Titin is part of the structural organization and assembly of the sarcomere from the Z-disc to the M-line along with other developmental, regulatory and mechanical functions in cardiac and skeletal muscle (Lee and Judge, 2017). Mutations in many sarcomeric proteins located at the Z-disc may also lead to cardiomyopathies (Knöll et al., 2002; Frank et al., 2006; Sheikh et al., 2007). For example, mutations in the myosin heavy chain 7 (*MYH7*) gene that encodes the sarcomeric protein β-Myosin Heavy Chain (β-MHC) cause PPCM (Walsh et al., 2010; Fiorillo et al., 2016; Bollen and van der Velden, 2017). β-MHC forms the heavy chain structure of type II myosin in sarcomeres, which in a sliding mechanism with actin filaments, generates the mechanical forces needed for muscle contraction. Mutations in the *STAT3* gene have also been found to contribute to PPCM (Ballard, 2019; Harhous et al., 2019). Other genes in which mutations have been reported to be associated with PPCM onset and progression include truncations in *DMD* (dystrophin that causes Duchenne’s Muscular Dystrophy) (Cheng and Prior, 2013; Ahmed et al., 2016), *DSP* (desmoplakin) (Ware et al., 2016), *TPM1* (α-tropomyosin)(Ware et al., 2016), and missense mutations in *MYBPC3* (cardiac myosin binding protein C) (Morales et al., 2010), *TNNC1* (cardiac troponin C) (Mestroni et al., 1994), *TNNT2* (cardiac troponin T) (Morales et al., 2010), and *LAMP2* (lysosome-associated membrane protein) (Ware et al., 2016). This list of gene mutations causing PPCM continues to grow as more genes are identified (Lee and Judge, 2017; Ballard, 2019). Additional evidence supporting an involvement of gene mutations in PPCM includes familial incidence, genome-wide association studies and variability of occurrence of PPCM among women from different regions and ethnicities (Lee and Judge, 2017).

### Metabolic Disorders and Dilated Cardiomyopathy

Metabolic cardiomyopathy is a heart muscle disorder that primarily develops in the presence of chronic metabolic conditions, such as type 2 diabetes, obesity, and insulin resistance (Figure 4; Nishida and Otsu, 2017). These conditions are frequently overlapping, resulting in similar metabolic-related structural and functional cardiac alterations, independent of hypertension or coronary artery disease and are collectively referred to as diabetic cardiomyopathy (Nakamura and Sadoshima, 2020). During the early stage of this disorder, metabolic disturbances do not cause significant structural changes in the heart, but result in other cellular abnormalities (e.g., impaired mitochondrial function, oxidative and ER stress and altered Ca^2+^ handling) all of which contribute to changes in diastolic function (Figure 4; Nishida and Otsu, 2017). However, as the disease progresses, these abnormalities accumulate, culminating in cardiomyocyte death, hypertrophy, fibrosis, and diastolic and systolic dysfunction (Riehle and Bauersachs, 2019; Tan et al., 2020; Figure 4). The etiology of metabolic cardiomyopathy is multifactorial and has been previously reviewed (Nishida and Otsu, 2017; Riehle and Bauersachs, 2019; Nakamura and Sadoshima, 2020; Tan et al., 2020).

In the presence of obesity, type 2 diabetes, or insulin resistance, the heart functions with a dysregulated energy metabolism. More specifically, impaired insulin-receptor signaling leads to reduced translocation of glucose transporter 4 to the cell membrane resulting in reduced glucose uptake and availability for oxidation (Figure 4; Cook et al., 2010; Tan et al., 2020). On the other hand, increased fatty acid uptake (Figure 4) and utilization occur due to increased membrane localization of fatty acid translocase (FAT/CD36) and higher PPAR-α activity (Coort et al., 2004; Nakamura et al., 2019; Riehle and Bauersachs, 2019), which induces the expression of genes involved in fatty acid uptake and oxidation and further prevents glucose oxidation through stimulation of pyruvate dehydrogenase kinase 4 expression (Nakamura and Sadoshima, 2020). Consequently, high rates of FAO and loss of glucose availability increase oxygen consumption, impair cardiac efficiency, and induce mitochondrial ROS production (Figure 4; Boudina et al., 2007; Borghetti et al., 2018). This imbalance between fatty acid uptake and oxidation leads to excessive accumulation of lipids and lipotoxic intermediates (e.g., ceramides) in cardiomyocytes, which has been associated with increased ROS levels, ER stress, mitochondrial membrane remodeling, and cardiomyocyte apoptosis (Figure 4; Bikman and Summers, 2011; Wende et al., 2012; Riehle and Bauersachs, 2019). Under hyperglycemic conditions, toxic glucose intermediates may contribute to the generation of advanced-glycosylation end products (AGEs) that trigger enhanced proinflammatory and profibrotic signaling in the heart (Singh et al., 2001; Nakamura and Sadoshima, 2020). These pathways promote increased extracellular matrix (ECM) protein production and reduced activity of ECM-degrading enzymes, both of which contribute to cardiac fibrosis and contractile dysfunction (Westermann et al., 2007; D’Souza et al., 2011; Borghetti et al., 2018). Additionally, activation of the renin-angiotensin-aldosterone system increases angiotensin II that stimulates cardiac fibrosis and hypertrophy (Kumar et al., 2012).

Despite the significant body of research reporting various possible mechanisms contributing to metabolic cardiomyopathy, the pathology of this disorder is still not entirely understood. Several studies have proposed that diabetes-induced changes in mitochondrial function lead to cardiomyopathy. A detailed review by Schilling (2015) discusses the hypothesis that diabetes promotes mitochondrial dynamic dysregulation, which is a trigger in the development of diabetic-induced cardiomyopathy progression and heart failure. However, more mechanistic studies are needed to confirm this hypothesis and understand the underlying pathobiology to advance treatment with targeted therapeutics. It should be noted that in a broader context, metabolic cardiomyopathy can also develop as a consequence of different inherited metabolic storage disorders that manifest during childhood (Albakri, 2019). However, their pathology differs from systemic disease-related cardiomyopathy and involves altered energy production due to deficiencies in certain enzymes regulating glycogen, glycolipid, and glycosaminoglycan metabolism (Guertl et al., 2001; Albakri, 2019).

### Mitochondrial-Associated Dilated Cardiomyopathy

Approximately 50% of patients suffering with mitochondrial diseases also present with cardiomyopathy (Florian et al., 2015). In many cases mitochondrial cardiomyopathies have an underlying genetic component resulting in malfunction of mitochondrial respiratory chain, FAO or cardiolipin synthesis and alterations of mitochondrial dynamics (El-Hattab and Scaglia, 2016a; Figure 4).

#### Dilated Cardiomyopathy Associated With OXPHOS Dysfunction

Taking into account that cardiac muscles are one of the high energy tissues in the body, it is not surprising that mitochondrial disorders associated with OXPHOS dysfunction manifest as cardiomyopathy (Figure 4; Thorburn et al., 2004). Moreover, mitochondrial disorder-related cardiomyopathies may be associated with defects in the synthesis of coenzyme Q10 (Potgieter et al., 2013), synthesis of the OXPHOS Fe–S clusters, transport of adenine nucleotides across IMM maintenance of mtDNA, transfer of mitochondrial RNAs, ribosomal proteins, ribosomal RNAs and translation factors (El-Hattab and Scaglia, 2016b). Mutations in the genes encoding mitochondrial proteins often lead to aberrant OXPHOS machinery resulting in not only an ATP deficiency but also increased ROS production and/or alterations in the antioxidant defense system, nitric oxide (NO) deficiency and dysregulation of Ca^2+^ homeostasis (El-Hattab and Scaglia, 2016a,b).

#### Dilated Cardiomyopathies Associated With Fatty Acid Oxidation Alterations

The energy substrates primarily used by the heart include fatty acids and carbohydrates; however fatty acids are the main energy substrate for the heart and they provide the majority of cofactors crucial for mitochondrial oxidative phosphorylation. All things considered, it is not surprising that alterations in the mitochondrial FAO pathway lead to the development of heart failure. Fatty acid and glucose metabolism are interconnected to regulate each other in a process referred to as the glucose/fatty acid cycle often called Randle Cycle (Randle et al., 1963; Arslanian and Kalhan, 1994). Interestingly, in the heart an increased rate of FAO decreases glucose oxidation and conversly an increased rate of glucose oxidation inhibits FAO (Figure 4). Alterations in key enzymes involved in FAO lead to mitochondrial cardiomyopathy. These enzymes include very long-chain acyl-CoA dehydrogenase (VLCAD), long-chain 3-hydroxyacyl-CoA dehydrogenase (LCHAD); trifunctional protein (TFP); carnitine-acylcarnitine translocase (CACT); carnitine palmitoyltransferase type 2 (CPT2); carnitine transporter (CT) and multiple acyl-CoA dehydrogenase (MAD) (Merritt et al., 2018). In the case of diabetic cardiomyopathy (where an oversupply of fatty acids is responsible for the observed cardiac lipotoxicity) excess fatty acids promote accumulation of lipid intermediates and surprisingly, in the case of diabetes, it is accompanied by increased FAO (Figure 4; Fillmore et al., 2014).

#### Dilated Cardiomyopathy Associated With Cardiolipin Synthesis Dysregulation

Cardiolipin is an essential constituent of IMM and contributes up to 20% of total IMM lipids (Schlame and Greenberg, 2017; Tatsuta and Langer, 2017). Due to the fact that IMM is comprised of approximately 75% protein and 25% lipid, alterations in the cardiolipin content and/or its composition have a direct impact on the structure and properties of the IMM and influence many mitochondrial processes including oxidative phosphorylation and protein translocation to mitochondria. Cardiolipin is essential (other phospholipids cannot substitute for it) for optimal function of the mitochondrial respiratory chain complexes I, III, and IV and is required for the structural integrity and formation of respiratory chain supercomplexes (Figure 4; Pfeiffer et al., 2003; Dudek et al., 2013; Letts et al., 2016). Moreover, changes in cardiolipin content or its’ species composition contribute to higher ROS production (Paradies et al., 2004; Nickel et al., 2014). Interestingly, mutations in the *DNAJC19* gene encoding a component of the mitochondrial protein import machinery in the IMM also induces cardiolipin accumulation with altered acyl chain. This is most likely due to DNAJC19’s interaction with prohibitins (PBH) to regulate cardiolipin remodeling (Richter-Dennerlein et al., 2014).

Barth Syndrome (BTHS) is a rare X-linked recessive mitochondrial cardiomyopathy caused by an altered cardiolipin metabolism. BTHS pathology includes changes in mitochondrial membrane phospholipids, lactic acidosis, organic aciduria and skeletal muscle weakness. BTHS is linked to gene mutations in the phospholipid transacylase localized to mitochondria, *taffazzin* (*TAZ)* (Dudek and Maack, 2017) and is involved in cardiolipin acyl chain remodeling. In physiological conditions up to 90% of cardiolipin in cardiac mitochondria exist as tetralinoleoylcardiolipin. Decreases in tafazzin activity reduce cardiolipin abundance and increase monolysocardiolipin levels. There are several detailed reviews on BTHS and cardiolipin metabolism in cardiomyopathy (Ikon and Ryan, 2017; see Dudek, 2017).

## Targeting Mitochondria to Attenuate Dilated Cardiomyopathy

Overall patients presenting with dilated cardiomyopathy continue to progress to end stage heart failure (1:3 patients) with disease maintenance occurring in 1:4 patients. Currently, a heart transplant is the best treatment option because there are no targeted therapies available due to the high complexity of dilated cardiomyopathy (Begic et al., 2018). Targeted therapies need to be developed that treat this complex disease potentially by targeting the underlying molecular pathways that are dysregulated. For example, targeting the OXPHOS pathway and subsequent mitochondrial dysfunction may be a way to attenuate cardiomyocyte death and maintain cardiac function (Brown et al., 2017). Advances in genetic analysis and molecular pathway alterations due to these gene mutations has paved the way for early detection and focused research on the development of potential preventative therapies (Verdonschot et al., 2019). Currently, mitochondrial-specific targeting therapies for dilated cardiomyopathy have not yet entered the clinic as they are at the basic research level. As research continues it will help determine whether restoring mitochondrial function may be way an efficacious way to treat dilated cardiomyopathies.

One research area that has emerged recently is the development of cardiac-specific organoids to study the complexities of cardiovascular disease. The term organoid is used to describe *in vitro* 3D multicellular tissues generated from pluripotent and adult stem cells that recapitulate some of the key structural and functional features of their *in vivo* counterparts (de Souza, 2018). Advances in efficient differentiation of induced-pluripotent cells (iPSC) into several cardiac cell types provide a novel unlimited cell source for developing cardiac organoids, especially due to the difficulties of adult stem cell isolation and ethical concerns regarding embryonic cells (Moretti et al., 2013; Zamani et al., 2018). Unlike traditional 2D cell cultures, cardiac organoids provide more accurate representations of the complexity of cell-cell and cell-extracellular matrix interactions. Moreover, being derived from human stem cells, they reflect cardiac (patho)physiology more appropriately compared with animal models thereby helping to overcome substantial functional between-species differences and animal to human translation issues (Moretti et al., 2013).

To date, iPSC cells and derived 3D organoids are used to model several cardiac disorders caused by cardiomyocyte gene mutations including dilated cardiomyopathy, hypertrophic cardiomyopathy, Barth’s syndrome and glycogen-storage cardiomyopathy (Wang G. et al., 2014; Hinson et al., 2015, 2016; Cashman et al., 2016; Nugraha et al., 2019). In addition to these monogenic diseases, cardiac organoids may provide great promise for modeling complex, lifestyle-related heart pathologies including acute myocardial infarction (Richards et al., 2020) and cardiomyopathy. For instance, relevant *in vitro* models of metabolic cardiomyopathy should embody all of the key features of the diabetic heart, including metabolic shift, lipotoxicity, insulin resistance and altered functionality (contractility and changes in mitochondrial function). Most of these characteristics have been previously recapitulated in 2D *in vitro* models that use iPSC-derived cardiomyocytes exposed to a diabetic-like environment (Drawnel et al., 2014). However, iPSC-derived cardiomyocytes are somewhat immature, with a fetal-like profile, which relies primarily on glucose utilization, compared to adult cardiomyocytes where metabolism depends on FAO. Ideally, a model of metabolic cardiomyopathy would provide evidence of this metabolic switch, which may be challenging to achieve using iPSC-derived cardiomyocytes (Jiang et al., 2018; Granéli et al., 2019). Nevertheless, considerable advances have been made in overcoming this issue of immaturity, especially using 3D culture, mechanical, or electrical conditioning techniques that provide a more adult-like cardiomyocyte phenotype in terms of oxidative metabolism, gene expression and calcium handling (Ronaldson-Bouchard et al., 2018; Golforoush and Schneider, 2020). Moreover, combining different cardiac cell types into 3D tissue-like organoids promotes the maturity and function of iPSCs-derived cardiomyocytes (Giacomelli et al., 2017; Golforoush and Schneider, 2020). Recently, cardiac organoids containing four different cell types (cardiomyocytes, epicardial, endothelial cells, and cardiac fibroblast) derived from the same iPSC have been created (Helms et al., 2019). Furthermore, Lee and colleagues developed a 3D heart organoid comprised of cardiomyocytes, conducting tissues, endothelial and smooth muscle cells, with atrial and ventricular parts, myocardial contraction, and gene expression profiles resembling their *in vivo* counterparts (Lee et al., 2020). Further research is necessary to determine how these organoids may be used to accurately model the complex pathology of dilated cardiomyopathy for a better understanding of underlying molecular mechanisms, mitochondrial function and identification of potential therapeutic targets.

## Conclusion

Mitochondria are the central hub of the cell, controlling and regulating several different functions such as bioenergetics, biosynthesis, ROS production and cell death. Considering the high-energy demand of the heart, much research has focused on how mitochondria play a central role in maintaining myocardial homeostasis. Morphological and functional changes of mitochondria are associated with CVD. Therefore, a finely tuned healthy mitochondrial network is required in order to prevent CVDs. Mechanisms of surveillance are also required to conserve mitochondrial fitness and alterations of this quality control system, which requires a delicate balance between fusion-fission machinery and mitophagy and loss of this balance, may lead to the accumulation of damaged and dysfunctional mitochondria. Loss of healthy mitochondria in the heart is linked directly with a decline of energy supply, increased ROS and culminates with cardiomyopathy onset and heart failure.

To date, current therapies focus on reducing heart energy demand and preventing further worsening of cardiac muscle function (Acquatella, 2000; Verdonschot et al., 2019). Continued research into the molecular mechanisms of CVD is needed to develop targeted therapies. Indeed, the continuing development of novel approaches such as organoids to study the aberrant pathology of the heart will further our understanding of how mitochondria are cardioprotective and identify new therapeutic targets for treating and preventing cardiomyopathies.

## Author Contributions

DR: conceptualization, writing—original draft, writing—review and editing. VM-U, FA, LM, YP, MW, IK, and MG: writing—original draft. PP: review and editing. CG: conceptualization, review, and editing. MM: conceptualization, funding acquisition, review, and editing. All authors have read and agreed to the published version of the manuscript.

## Conflict of Interest

The authors declare that the research was conducted in the absence of any commercial or financial relationships that could be construed as a potential conflict of interest.
